# Special Issue “New Trends in Diabetes, Hypertension and Cardiovascular Diseases: 3rd Edition”

**DOI:** 10.3390/ijms262110536

**Published:** 2025-10-29

**Authors:** Yutang Wang, Dianna J. Magliano

**Affiliations:** 1 Discipline of Life Science, Institute of Innovation, Science and Sustainability, Federation University Australia, Ballarat, VIC 3350, Australia; 2 Diabetes and Population Health, Baker Heart and Diabetes Institute, Melbourne, VIC 3004, Australia

## 1. Introduction

Cardiovascular disease (CVD) encompasses a broad spectrum of conditions affecting the heart and blood vessels [1,2,3,4,5]. It remains the leading cause of death globally, responsible for approximately 19.8 million deaths in 2022—accounting for 32% of the total global mortality [6]. Beyond its profound health impact, CVD also imposes a significant economic burden. In the United States alone, annual costs were estimated at USD 393 billion in 2020, with projections reaching USD 1.49 trillion by 2050 [7,8]. These figures highlight the urgent need for cost-effective strategies to promote cardiovascular health, reduce healthcare expenditures, and improve population outcomes [8,9].

Most CVD cases are regarded preventable through the modification of behavioral and environmental risk factors [6]. Major contributors include unhealthy dietary patterns [10,11,12], tobacco use [13,14], excessive alcohol consumption [15], physical inactivity [16,17], obesity [18], and exposure to polluted air [19,20]. These findings suggest that implementing behavioral change programs targeting these modifiable factors could play a critical role in reducing the burden of CVD. For instance, dietary interventions that emphasize a healthy eating pattern [21], rich in fruits and vegetables [22], and low in saturated fats [23], sodium, cholesterol [24], processed meats [25], and trans fats [26], may significantly lower CVD risk. Additionally, regular physical activity is another key strategy for CVD prevention [27,28].

Despite the availability of various behavioral modification programs, medications, and surgical interventions [21,29,30,31], CVD continues to be the leading cause of morbidity and mortality. This underscores the importance of further investigating the pathogenesis of CVD and developing novel therapies and therapeutic agents [32,33,34,35], leveraging cellular [36], tissue [37], and animal models [38,39,40,41].

According to the World Health Organization, hypertension affects 33% of the adult population, with 44% of individuals unaware of their condition [42]. Additionally, 14% of adults aged ≥18 years are living with diabetes, and 59% of diabetic adults aged ≥30 years are not receiving medication for their condition [43]. Both hypertension and diabetes are independent risk factors for CVD [44,45,46,47]. Therefore, controlling blood pressure and blood glucose levels is essential for reducing CVD risk [48,49,50].

According to the latest Global Burden of Disease (GBD) study [51], high blood pressure, particulate matter pollution, and high blood glucose are the top three risk factors contributing to the global disease burden. The Disability-Adjusted Life Year (DALY), an age-standardized metric combining years of life lost due to premature death and years lived with disability, is commonly used to assess overall health burden [52,53]. High blood pressure, particulate matter pollution, and high blood glucose account for 8.4%, 8.2%, and 5.8% of total DALYs, respectively [51]. Therefore, controlling blood pressure and blood glucose levels would significantly improve the population health.

Triglycerides are also recognized as a risk factor for hypertension [54,55], diabetes [56], and CVD [57,58]. Consequently, lowering circulating triglyceride levels may further contribute to CVD risk reduction [59,60].

## 2. Contribution of This Special Issue to the Field of Research

This Special Issue features five review articles [61,62,63,64,65] and three original research papers [66,67,68], collectively showcasing recent advances in the fields of diabetes, hypertension, and CVD. These contributions provide valuable insights into ongoing progress and emerging trends across these domains (Table 1). Together, the articles address a broad spectrum of topics, including the following:

- Risk factors, such as the role of gut microbiota [61,68];
- Disease pathogenesis, encompassing mechanisms like macrophage activation [63], sympathetic nervous system stimulation [65], and genetic influences [67];
- Therapeutic targets, including aldehyde dehydrogenase 2 [62] and apurinic/apyrimidinic endonuclease 1 [64]; and;
- Treatment strategies, such as the application of docetaxel [66].

Readers are encouraged to explore these articles to gain a deeper understanding of the findings and their implications for future research and clinical practice.

**Table 1:** Summary of the eight articles in this Special Issue.

References	Title	Authors	Main Finding
Reviews			
[61]	Shared Risk Factors and Molecular Mechanisms Between Aortic Stenosis and Atherosclerosis: A Rationale for Therapeutic Repositioning	Cinezan C, Magureanu DC, Hiceag ML, et al.	This article examines the shared risk factors and molecular mechanisms of aortic stenosis and atherosclerosis. It reveals that both diseases are driven by age, hypertension, hyperlipidemia, and diabetes, and involve chronic inflammation, endothelial dysfunction, oxidative stress, lipid accumulation, and calcific remodeling.
[62]	ALDH2 Enzyme Deficiency in Diabetic Cardiomyopathy	Hsieh Y-W, Lee A-S, Sung K-T, et al.	This study reviews the molecular basis of how ALDH2 enzyme deficiency contributes to diabetic cardiomyopathy. The article highlights promising therapeutic strategies, including the use of ALDH2 activators and SGLT2 inhibitors, to mitigate disease progression.
[63]	Tissue-Resident Macrophages in Cardiovascular Diseases: Heterogeneity and Therapeutic Potential	An T, Guo M, Wang Z, et al.	This article explores the origin, diversity, and roles of cardiac resident macrophages in cardiovascular conditions such as myocardial infarction, heart failure, atherosclerosis, and hypertension. It emphasizes the therapeutic potential of targeting specific macrophage subtypes to modulate disease outcomes.
[64]	From DNA Repair to Redox Signaling: The Multifaceted Role of APEX1 (Apurinic/Apyrimidinic Endonuclease 1) in Cardiovascular Health and Disease	Yuan H-H, Yin H, Marincas M, et al.	This study highlights the role of APEX1 in maintaining cardiovascular homeostasis through regulation of innate immunity. The findings suggest APEX1 as a novel therapeutic target for treating various cardiovascular diseases.
[65]	Neuroimmune Interactions and Their Role in Immune Cell Trafficking in Cardiovascular Diseases and Cancer	Wang Y, Anesi JC, Panicker IS, et al.	This article outlines key molecular pathways activated by sympathetic nervous system stimulation and their impact on immune cell migration. It proposes that inhibiting sympathetic activity could be a viable therapeutic approach for both cardiovascular disease and cancer.
Original research articles			
[66]	Low-Dose Docetaxel Is Effective in Reducing Atherogenic Lipids and Atherosclerosis	Choi HY, Ruel I, Choi S, et al.	This study investigates the effect of docetaxel, a compound that enhances high-density lipoprotein biogenesis, on atherosclerosis in apolipoprotein E-deficient mice. The study shows that docetaxel reduces dyslipidemia-induced atherosclerosis, supporting its potential as an HDL-targeted therapy.
[67]	The Genetic Variants Influencing Hypertension Prevalence Based on the Risk of Insulin Resistance as Assessed Using the Metabolic Score for Insulin Resistance (METS-IR)	Shine B-K, Choi J-E, Park Y-J, et al.	This article utilizes data from the Korean Genome and Epidemiology Study and identifies six genetic loci significantly associated with hypertension. It reveals distinct genetic patterns across metabolic profiles, highlighting the interplay between metabolic status and genetic predisposition.
[68]	Gut Microbiota and Metabolic Alterations Associated with Heart Failure and Coronary Artery Disease	Yafarova AA, Dementeva EV, Zlobovskaya OA, et al.	This study assesses the role of gut microbiota in cardiovascular disease, identifying microbial features linked to coronary artery disease and heart failure. The findings underscore complex host–microbiome interactions that may influence cardiovascular health and disease progression.

ALDH2, Aldehyde dehydrogenase 2; APEX1, apurinic/apyrimidinic endonuclease 1; HDL, high-density lipoprotein; SGLT2, sodium-glucose cotransporter 2.

## 3. Concluding Remarks

This Special Issue presents recent advancements in the research areas of diabetes, hypertension, and CVD. We hope these findings will contribute meaningfully to reducing the morbidity and mortality associated with these conditions. Emerging approaches such as machine learning [69,70], precision medicine [71,72], and early biomarker identification [73,74], along with the implementation of lifestyle modification strategies for CVD prevention in the general population [75,76], represent promising directions for future research and risk reduction.
